# Serum and gene expression profile of cytokines in first-episode psychosis

**DOI:** 10.1016/j.bbi.2012.06.010

**Published:** 2013-07

**Authors:** Marco Di Nicola, Annamaria Cattaneo, Nilay Hepgul, Marta Di Forti, Katherine J. Aitchison, Luigi Janiri, Robin M. Murray, Paola Dazzan, Carmine M. Pariante, Valeria Mondelli

**Affiliations:** a King’s College London, Institute of Psychiatry, Department of Psychological Medicine, London, UK; b King’s College London, Institute of Psychiatry, Department of Psychosis Studies, London, UK; c The South London and Maudsley NHS Foundation Trust & Institute of Psychiatry NIHR Biomedical Research Centre for Mental Health, London, UK; d King’s College London, Institute of Psychiatry, MRC SGDP Centre, London, UK; e Department of Biomedical Sciences and Biotechnologies, Biology and Genetic Division, Brescia University School of Medicine, Brescia, Italy; f Department of Neuroscience, Institute of Psychiatry and Psychology, Catholic University of Sacred Heart, Rome, Italy

**Keywords:** Cytokine, PBMC, Psychosis, Schizophrenia, Stress, Childhood trauma, Gene expression, Inflammation, Nicotine, Cannabis

## Abstract

An inflammatory syndrome has been previously reported in chronic schizophrenia. The aims of this study were to investigate: (1) serum levels and leukocyte gene expression of cytokines in patients with first-episode psychosis and controls; and (2) possible causes of abnormal cytokine levels in first-episode psychosis, testing their association with psychosocial stressors, current nicotine and cannabis use, and duration of antipsychotic treatment. We recruited 24 first-episode psychosis patients and 24 healthy controls matched for age, gender, ethnicity and body mass index. Serum interleukin(IL)-1α, IL-1β, IL-2, IL-4, IL-6, IL-8, IL-10, Tumour Necrosis Factor- α (TNF-α), Interferon- γ (IFN-γ), vascular endothelial growth factor (VEGF), epidermal growth factor (EGF), and monocyte chemotactic protein-1 (MCP-1) were analysed in all subjects. Leukocyte gene expression analyses were conducted only for those cytokines that were different between-groups in the serum analyses. Patients had significantly higher serum levels of IL-1α (effect size *d* = 0.6, *p* = 0.03), IL-1β (*d* = 0.4, *p* = 0.01), IL-8 (*d* = 0.6, *p* = 0.01) and TNF-α (*d* = 0.7, *p* = 0.05) and a trend for higher IL-6 serum levels (*d* = 0.3, *p* = 0.09) when compared with controls. Leukocyte m-RNA levels of IL-1α (*d* = 0.6, *p* = 0.04), IL-6 (*d* = 0.7, *p* = 0.01) and TNF-α (*d* = 1.6, *p* < 0.001), but not IL-1β and IL-8, were also significantly higher in patients. A history of childhood trauma was associated with higher TNF-α serum levels (*p* = 0.01), while more recent stressful life-events were associated with higher TNF-α mRNA levels in leukocytes (*p* = 0.002). In conclusion, first-episode psychosis is characterised by a pro-inflammatory state supported, at least in part, by activation of leukocytes. Past and recent stressors contribute to this pro-inflammatory state.

## Highlight

► First-episode psychosis is characterised by a pro-inflammatory state supported partly by activation of leukocytes. Stress contributes to this pro-inflammatory state.

## 1 Introduction

In the past two decades, several studies have investigated the relevance of cytokine alterations in the multifactorial pathogenesis of schizophrenia (Erbagci et al., 2001; Cazzullo et al., 2001; Zhang et al., 2002; Na and Kim, 2007). A recent meta-analysis of these studies suggests the presence of an inflammatory syndrome in schizophrenia, with some cytokines mainly present during acute exacerbations and possibly to be considered as state marker for acute schizophrenia, such as interleukin (IL)-1β, IL-6 and transforming growth factor- β (TGF-β), and other cytokines remaining elevated following antipsychotic treatment and possibly to be considered as trait markers, such as IL-12, interferon-γ (INF-γ) and tumor necrosis factor-α (TNF-α) (Miller et al., 2011). Cytokine alterations in patients with chronic schizophrenia may be partly due to weight gain, metabolic disorders and obesity (Henderson, 2005), long duration of illness, or multiple drug treatment (Drzyzga et al., 2006). In order to reduce the role of these possible confounding factors, it is important to study cytokine network alterations in subjects with a first-episode of psychosis. First-episode psychosis subjects have a short exposure to antipsychotic drugs, with fewer metabolic effects and immunological changes associated to these drugs. To date, only few studies have investigated cytokine levels in subjects with first-episode psychosis, reporting higher serum or plasma levels of IL-1β, IL-6, IL-12, INF-γ, TNF- α, TGF- β and soluble IL-2 receptor (Ganguli and Rabin, 1989; Kim et al., 2000, 2002, 2004, 2009; Theodoropoulou et al., 2001; Crespo-Facorro et al., 2008; Fernandez-Egea et al., 2009; Song et al., 2009).

Some authors have suggested that high levels of pro-inflammatory cytokines in psychosis could be related to an over-activation of the Th1 activity (Crespo-Facorro et al., 2008), resulting in an imbalance in the Th1/Th2 cytokines ratio, with a shift towards the Th2 system (Schwarz et al., 2001). However, variations in serum or plasma cytokines levels might not directly reflect the activity of peripheral blood immune cells. Indeed, serum and plasma cytokine levels may partly derive from the vessel walls or from other lymphoid or non-lymphoid cells, such as hepatocytes or adipocytes (O’Rourke et al., 2006). For these reasons, studying cytokine gene expression in peripheral blood cells might represent a more reliable method to investigate the specific activity of immune cells and their degree of activation. Moreover, leukocytes have been proposed as a useful peripheral model to study inflammatory processes in the central nervous system (Sullivan et al., 2006). In fact, their expression profiles have shown similarities to those observed in brain cells, especially for genes encoding stress mediators, cytokines, hormones, and growth factors (Glatt et al., 2005; Sullivan et al., 2006).

In order to capture a comprehensive picture of the inflammatory pattern in first-episode psychosis, we focussed on a wide range of inflammatory markers, including pro-inflammatory cytokines (interleukins IL-1α, IL-1β, IL-2, IL-6, interferon-gamma (IFN-γ), tumor necrosis factor-alpha (TNF-α)), anti-inflammatory cytokines (IL4, IL-10), chemokines (IL-8, monocyte chemotactic protein-1 (MCP-1)), and grow factors (vascular endothelial growth factor (VEGF), epidermal growth factor (EGF)). The first aim of this study was to investigate differences between first-episode psychosis subjects and healthy volunteers in the serum; cytokines found to be different in serum were then investigated as leukocyte gene expression. The second aim of the study was to understand the possible causes of altered cytokine serum and gene expression levels in first-episode psychosis, testing their association with psychosocial stress, current nicotine and cannabis use, and duration of antipsychotic treatment. Patients and controls were matched on clinical and socio-demographic variables, including ethnicity and body mass index (BMI), to reduce potential confounders.

## 2 Methods

### 2.1 Subjects

Twenty-four patients (16 males and 8 females; mean age ± SEM: 28.1 ± 1.1 years) presenting with a first-episode of a functional psychosis were recruited from the South London and Maudsley (SLAM) NHS Foundation Trust (Mondelli et al., 2010a,b). Patients with organic psychosis, learning disabilities or requiring a translator because of lack of English fluency were excluded from the study. Twenty-four healthy subjects (15 males and 9 females; mean age: 26.6 ± 0.9 years) were also recruited. Patients and controls did not significantly differ for age (*p* = 0.3), gender (*chi square* = 0.09, *p* = 1.0), ethnicity (*p* = 0.2) and BMI (*p* = 0.1). Two patients reported some physical health issues, one reporting a diagnosis of sickle cell disease and the second one reporting history of asthma (not under any specific medication at time of assessment). The study was approved by the local Ethics Committee and written informed consent was obtained from all participants.

Validation of clinical diagnosis according to DSM-IV criteria (American Psychiatric Association, 2000) was obtained using the Operational Criteria (OPCRIT) (McGuffin et al., 1991). Ten patients had a diagnosis of schizophrenia-like disorder, 9 of affective psychosis and five of psychotic disorder not otherwise specified. The mean duration of antipsychotic treatment was 33.5 ± 7.2 days (range 0–129 days). Sixteen patients were taking atypical antipsychotics (such as olanzapine and risperidone), 3 haloperidol, and 5 were drug naïve. Blood samples were collected in all subjects in non-fasting conditions using clot activator tubes for serum analysis and PaxGene tubes for gene expression analysis.

### 2.2 Stress measurements

We collected information about stressful life events which had occurred in the previous six months using the Brief Life Events questionnaire (Brugha and Cragg, 1990), and measured perceived stress in the previous month using the Perceived Stress Scale (Cohen and Williamson, 1988). Information about childhood trauma were also collected, using a modified version of the Childhood Experience of Care and Abuse (CECA) Questionnaire (Bifulco et al., 2005), including information about loss of parents, separation from parents for more than 6 months, and physical and sexual abuse before the age of 17 years.

### 2.3 Cytokines serum analysis

The serum was separated, aliquoted and stored at −70 °C before use. Biochip array technology was used to perform simultaneous quantitative detection of multiple analytes from a single patient sample. The core technology is the Randox Biochip (http://www.randox.com), a solid-state device containing an array of discrete test regions of immobilised antibodies specific to different cytokines and growth factors. A sandwich chemiluminescent immunoassay was employed for the cytokine array. This cytokine array measures the following cytokines and growth factors: interleukin (IL)-1α, IL-1β, IL-2, IL-4, IL-6, IL-8, IL-10, vascular endothelial growth factor (VEGF), Tumour Necrosis Factor- α (TNF-α), Interferon- γ (IFN-γ), epidermal growth factor (EGF), monocyte chemotactic protein-1(MCP-1). Intra-assay precision of the cytokine array is shown in Table 1. Sensitivity of cytokine assay is shown in Table 2.

### 2.4 Gene expression analysis

Gene expression analyses were conducted only for cytokines found to be different in the serum when compared between patients and controls. PaxGene Tubes were collected at the same time of the serum samples and kept for two hours at room temperature after blood samples were withdrawn and then stored at −80 °C until they were processed. RNA isolation was performed using the PAXGene Blood RNA Kit (Qiagen S.p.A., Milan, MI, Italy) according to manufacturer’s protocols. Two micrograms of total RNA were used for cDNA synthesis and for subsequent gene expression analysis in Real Time PCR. Quantitative Real-Time PCR was performed using HOT FIREPol® EvaGreen® qPCR Mix (Solis BioDyne, Tartu, Estonia) according to the SYBR Green method. For each target primer set, a validation experiment was performed to demonstrate that PCR efficiencies were within the range of 90–100% and equal to the efficiencies of the reference genes (glyceraldehyde 3-phosphate dehydrogenase (GAPDH), beta-actin (ACTB) and beta-2-microglobulin (B2M)). Each sample was assayed in duplicate and each target gene (IL-1α, IL-1β, IL-6, IL-8 and TNF-α) was normalized to the gene expression of the three reference genes: GAPDH, ACTB and B2M. Data were normalized to the geometric mean of all three reference genes and expressed as Relative Expression Ratio (R).

### 2.5 Data analysis

Data were analyzed using the Statistical Package for Social Sciences, Version 15.0 (SPSS Inc.). Continuous variables are presented as mean ± standard error mean. Independent *T*-test and Chi-square test were used to compare respectively continuous and categorical variables between groups. Parametric and non-parametric correlation analyses were used, as appropriate, to test association between cytokine levels and measures of psychosocial stress, antipsychotic treatment and current nicotine and cannabis use, separately in patients and controls. Serum cytokine levels were normalized for the statistical analyses through logarithmic transformation. Serum cytokines levels are presented as raw values, while the statistics was conducted on the logarithmic transformed values.

## 3 Results

First-episode psychosis patients showed significantly higher serum levels of IL-1α (effect size *d* = 0.6), IL-1β (*d* = 0.4), IL-8 (*d* = 0.6) and TNF-α (*d* = 0.7) compared with healthy controls. Patients also had a trend for higher IL-6 serum levels (*d* = 0.3). No significant difference was found between patients and controls for other serum cytokines (Table 3). We did not find any significant correlation between BMI or duration of antipsychotic treatment and cytokine levels in our patients’ sample (*p* > 0.05).

Gene expression analyses conducted only in those cytokines that were different in the serum (IL-1α, IL-1β, IL-6, IL-8 and TNF-α) revealed significantly increased mRNA levels of IL-1α (*d* = 0.6), IL-6 (*d* = 0.7) and TNF-α (*d* = 1.6) in first-episode psychosis patients when compared with controls. No significant difference in gene expression of IL-1β and IL-8 was found between patients and controls (Table 3). When looking at correlations between serum levels and mRNA levels of each of the above cytokines, we found a significant correlation between serum and mRNA levels only for IL-6 (*r* = 0.553, *p* = 0.005), and a trend for TNF-α (*r* = 0.375, *p* = 0.07); no significant correlation was found between serum and mRNA levels for the other cytokines (IL-1α, IL-1 β, IL-8).

In order to assess the Th1/Th2 cytokines balance between subjects with first-episode psychosis and healthy controls, we calculated the IFNγ/IL-4 ratio. We did not find a significant difference between the two groups (first-episode psychosis vs controls: 0.56 ± 0.06 vs 0.95 ± 0.56, *t* = 0.7, *p* = 0.5).

### 3.1 Associations between cytokines levels and psychosocial stress

When looking at the effect of psychosocial stress on cytokine levels, we found that perceived stress was significantly positively correlated with IL-6 mRNA levels in the control group (*r* = 0.524, *p* = 0.02), and with INF-γ serum levels in the patients’ group (*r* = 0.486, *p* = 0.02). We also found that the number of recent stressful life events was significantly positively correlated with mRNA levels of IL-1β (*ρ* = 0.468, *p* = 0.04) and of TNF-α (*ρ* = 0.661, *p* = 0.002) in the patients’ group. When looking at the effect of childhood trauma, we found that healthy controls with childhood trauma had significantly higher mRNA levels of IL-1β compared with healthy controls without childhood trauma (1.5 ± 0.2 vs 0.9 ± 0.1, *t* = −2.6, *p* = 0.02). Similarly, patients with childhood trauma had significantly higher serum levels of TNF-α (88.7 ± 25.5 vs 7.2 ± 1.0 pg/ml, *t* = −3.2, *p* = 0.006) and MCP-1 (216.7 ± 24.9 vs 62.4 ± 9.4 pg/ml, *t* = −4.3, *p* = 0.001), but lower serum levels of VEGF (120.8 ± 40.6 vs 165.0 ± 37.7 pg/ml, *t* = 3.0, *p* = 0.008) when compared with patients without childhood trauma. We did not find any other significant association with other cytokines (serum or mRNA levels) in either patients’ or control group.

### 3.2 Associations between cytokines levels and current nicotine or cannabis use, and employment

We found that serum levels of IL-6 were significantly higher in patients smoking nicotine than in patients who did not smoke (3.2 ± 1.0 vs 1.0 ± 0.2 pg/ml, *t* = 2.2, *p* = 0.04). We also found that patients smoking cannabis had significantly lower serum levels of IL-2 (3.4 ± 0.7 vs 19.7 ± 14.6 pg/ml, *t* = −2.1, *p* = 0.046) and mRNA levels of IL-1α (1.9 ± 0.4 vs 3.6 ± 0.6, *t* = −2.1, *p* = 0.048), but higher serum levels of MCP-1 (240.3 ± 34.1 vs 137.6 ± 19.1 pg/ml, *t* = 2.5, *p* = 0.02) when compared with patients who did not use cannabis. We did not look at the effect of cannabis on cytokines levels in healthy controls, since only two of our controls were current cannabis users.

When looking at the possible effect of employment on cytokines levels, we found lower mRNA levels of IL-8 in unemployed healthy controls when compared with employed healthy controls (1.1 ± 0.2 vs 0.7 ± 1.1, *t* = 2.1, *p* = 0.048). Patients who were unemployed had significantly higher serum levels of IL-4 (3.6 ± 0.3 vs 2.4 ± 0.2, *t* = −2.8, *p* = 0.01), VEGF (143.6 ± 29.7 vs 58.5 ± 57.8, *t* = −3.0, *p* = 0.007), and EGF (256.4 ± 34.0 vs 123.4 ± 38.4, *t* = −3.0, *p* = 0.008), and lower serum levels of TNF-α (46.1 ± 22.2 vs 123.3 ± 26.5, *t* = 2.8, *p* = 0.01).

## 4 Discussion

Our findings confirm the presence of an inflammatory syndrome in patients with first-episode of psychosis, in the absence of Th1/Th2 ratio changes.

Interestingly, the finding of elevated levels of IL-1α, IL-6 and TNF-α, both in serum and leukocytes gene expression analyses, provides direct evidence that the immune cells are affected in psychosis, and suggests that the peripheral blood immune cells are the main source of these cytokines’ elevated levels in the serum. In contrast, IL-1β and IL-8 were elevated in the serum but not in leukocyte gene expression analyses. This is in agreement with other studies finding discrepancies between serum cytokine levels and cytokine mRNA levels in peripheral blood mononuclear cells (PBMCs) in other populations (O’Rourke et al., 2006). This difference may be the result of abnormalities in post-transcriptional regulation of cytokine expression, leading to a lack of correlation between transcript levels and protein levels. Moreover, gene expression may be more easily and rapidly affected than systemic cytokine levels (Futh et al., 2004), and therefore some differences between gene expression and serum levels might be related to the effects of more recent and acute vs chronic activation of the immune system. Alternatively, peripheral blood immune cells are not the primary source of IL-1β and IL-8 elevated serum levels in these patients. Indeed, elevated cytokines may derive from cytokine-producing cells other than peripheral immune cells, such as intra-hepatic or adipose tissue-associated cells (O’Rourke et al., 2006). In our study, we matched our patients and controls for body mass index, in order to minimize the effect on a potential excess of abdominal adipose tissue on inflammatory markers in patients. However, individuals with identical BMI may have different body compositions with regard to fat and muscle, so we cannot exclude a role of the adipose tissue or of other lymphoid or non-lymphoid cells in contributing to the elevated levels of IL-1β and IL-8 found in our patients. Indeed, increased intra-abdominal fat deposition has been previously reported in depressed patients who did not differ for BMI from the comparison healthy control group (Thakore et al., 1997).

Interestingly, the fact that cytokine expression differs between the serum and gene expression analyses is also reflected in the pattern of associations between serum or leukocytes mRNA cytokines levels and psychosocial and clinical factors. For example, both serum and gene expression levels of TNF-α are elevated in patients, but only serum levels correlate with a history of childhood trauma. This could be again explained by the effects of a long-lasting chronic activation of the immune system, affecting serum levels. This notion is consistent with our previous studies finding an association between a history of childhood trauma and peripheral inflammation in adults (Danese et al., 2007, 2008) as well as children (Danese et al., 2010). In contrast, only leukocytes mRNA levels correlate with a higher number of recent life events, suggesting that more recent stressors are needed to activate immune cells.

The presence of a pro-inflammatory state at the onset of psychosis supports the role of increased inflammation in the development of psychosis. Indeed, previous studies have shown that cytokines can affect the synthesis, release and reuptake of neurotransmitters as well as influence neurogenesis (Miller et al., 2009; Raison and Miller, 2011; Zunszain et al., 2012a,b). In particular, it has been recently shown that IL-1β exerts a negative effect on human hippocampal neurogenesis, by activating the kynurenine pathway and subsequently affecting the availability of tryptophan and the production of enzymes conducive to neurotoxic metabolites (Zunszain et al., 2012a). Moreover, preclinical studies have suggested that pro-inflammatory cytokines could directly reduce gene transcription of brain-derived neurotrophic factor (BDNF), further supporting a role of inflammation in influencing neuroplasticity (Murphy et al., 2000). Interestingly we have recently shown that stressful events can influence BDNF expression at the onset of psychosis, and that this association is possibly mediated by an inflammatory pathway (Mondelli et al., 2011).

Our preliminary findings support the hypothesis that childhood trauma and recent stressors contribute to the pro-inflammatory state shown in patients with psychosis. In particular, we found that childhood trauma and recent stressors increase levels of pro-inflammatory cytokines (TNF-α, IL-1β, INF-γ, IL-6) and chemokines (MCP-1) in either patients or controls, and decrease levels of the growth factor VEGF in the patients’ group. It is unclear why psychological stress would affect specific cytokines without apparently affecting others, or why stress would affect different cytokines in patients and in healthy controls. Indeed, the association between childhood trauma and increased levels of TNF-α has been already recently reported in patients with schizophrenia (Dennison et al., 2012), while this is the first time that childhood trauma has been found to be associated with increased levels of MCP-1 and decreased levels of VEGF in patients with psychosis. MCP-1 is a pro-inflammatory chemokine known to play a major role in the development of cardiovascular disease by promoting the recruitment of leuokocytes to sites of inflammation in the vessel wall (Hansson and Hermansson, 2011). On the other hand, VEGF is known to have neurotrophic and neuroprotective actions; low serum VEGF levels have been associated with Alzheimer’s disease, suggesting their role in a possible neurodegenerative process (Mateo et al., 2007). Indeed, further studies would need to confirm these preliminary results and further investigate whether, and why, psychological stress affects only specific cytokines.

Our exploratory analyses also showed an association between current nicotine use and higher IL-6 serum levels but not IL-6 mRNA levels, suggesting that the increased IL-6 production by the PBMCs cannot be explained by current nicotine use. Cigarette smoking has been recently shown to have selective effects on serum components of patients with schizophrenia that lead to altered immune function different from healthy controls (Herberth et al., 2010). This might partly explain the specific effect of nicotine use on IL-6, and the lack of its effect on cytokine levels in our group of healthy controls. Our findings of lower IL-1α and IL-2 levels associated with current cannabis use are not surprising, and are consistent with previous pre-clinical and clinical studies showing an anti-inflammatory effect of cannabinoids (Klein and Cabral, 2006). Interestingly, we also found that cannabis use was associated with higher levels of MCP-1 in our patients. One previous study has demonstrated that a cannabinoid agonist induces a modulation of specific chemokines, such as IL-8 and MCP-1, but not of other chemokines (GRO-α and MIP-1α), indicating a possible specific molecular targeting of cannabinoid agonists (Jbilo et al., 1999).

Some limitations of the study need to be acknowledged. First, for the second aim of the study we conducted multiple correlation analyses in a relatively small sample of subjects. However, these should be considered explorative analyses, reporting preliminary findings which will need to be validated by future studies. Secondly, our subjects were non-fasting at time of blood sampling and the time of blood sampling was random during the day. However, patients and controls did not appear to differ significantly for time of blood sampling, and the blood sample for serum and mRNA levels was taken at the same time point for each subject. Moreover, our group of patients was heterogeneous for diagnosis, since it included both patients with schizophrenia-like and affective psychosis. However, a diagnostic heterogeneity is typical in first-episode psychosis, since patients are at the beginning of their illness, and it represents better what we would expect in a clinical setting. Last, most patients were taking antipsychotic treatment, although for a short period of time, and this could have confounded some of our findings. However, we did not find a correlation between duration of antipsychotic and cytokine levels in our patients.

In conclusion, first-episode psychosis patients are characterised by a pro-inflammatory state that appears mainly mediated by activation of the PBMCs; however, other peripheral tissues (such as hepatic or adipose tissue) also seem to contribute to this inflammatory syndrome. Stressful events, both in childhood and closer to psychosis onset, appear to contribute to the increased cytokine production in first-episode psychosis.

## Conflict of interest statement

All authors declare that there is no conflict of interest.

## Figures and Tables

**Table 1:** Intra-assay precision of cytokine array.

Analyte	Level 1		Level 2		Level 3	
	Conc. (pg/mL)	%CV	Conc. (pg/mL)	%CV	Conc. (pg/mL)	%CV
IL-2	32.0	9.6	187	5.0	506	6.3
IL-4	22.0	10.7	165	9.4	388	4.6
IL-6	2.2	12.9	21	6.1	44	8.7
IL-8	22.0	10.1	292	7.5	1056	7.9
IL-10	18.0	6.3	214	5.5	457	6.7
VEGF	30.0	7.3	317	4.6	721	4.3
IFN	15.0	12.7	274	6.2	1047	3.8
TNF-α	15.7	10.2	233	6.0	768	7.8
IL-1α	4.9	9.7	105	7.7	339	6.7
IL-1β	10.6	8.2	139	6.3	406	6.7
MCP-1	60.0	6.8	297	5.1	512	4.7
EGF	60.0	4.6	173	5.8	339	7.6

**Table 2:** Sensitivity of cytokine assay.

Analyte	Calibration range (pg/mL)	Sensitivity (pg/mL)
IL-2	0–3000	4.8
IL-4	0–4000	6.6
IL-6	0–900	1.2
IL-8	0–3000	7.9
IL-10	0–1000	1.8
VEGF	0–3000	14.6
IFN	0–1500	3.5
TNF-α	0–1500	4.4
IL-1α	0–500	0.8
IL-1β	0–500	1.6
MCP-1	0–1500	13.2
EGF	0–900	2.9

**Table 3:** Cytokines serum levels and cytokine gene expression in patients with first-episode psychosis and controls. Serum cytokines levels are presented as raw values, while the statistics was conducted on the logarithmic transformed values. Statistically significant values are presented in bold.

	Patients	Controls	Statistics
IL-1α (pg/ml)	1.9 ± 0.3	1.1 ± 0.3	*t* = −2.2, *p* = **0.03**
IL-1β (pg/ml)	6.0 ± 2.8	2.1 ± 0.4	*t* = −2.7, *p* = **0.01**
IL-2 (pg/ml)	13.6 ± 9.2	4.5 ± 1.3	*t* = −1.4, *p* = 0.2
IL-4 (pg/ml)	3.4 ± 0.3	4.6 ± 0.8	*t* = 1.6, *p* = 0.1
IL-6 (pg/ml)	2.4 ± 0.6	1.5 ± 0.5	*t* = −1.8, *p* = 0.09
IL-8 (pg/ml)	435.4 ± 170.7	71.9 ± 21.8	*t* = −2.6, *p* = **0.01**
IL-10 (pg/ml)	1.5 ± 0.2	1.9 ± 0.6	*t* = −0.6, *p* = 0.5
TNF-α (pg/ml)	62.1 ± 17.7	15.9 ± 5.0	*t* = −2.0, *p* = **0.05**
INF-γ (pg/ml)	1.8 ± 0.2	3.7 ± 1.8	*t* = −0.7, *p* = 0.5
VEGF (pg/ml)	123.4 ± 25.3	182.1 ± 32.0	*t* = 1.7, *p* = 0.1
EGF (pg/ml)	228.1 ± 27.8	227.3 ± 19.0	*t* = 0.3, *p* = 0.8
MCP-1 (pg/ml)	176.1 ± 19.9	156.3 ± 19.0	*t* = −0.7, *p* = 0.5
IL-1α mRNA (R)	3.0 ± 0.4	1.7 ± 0.4	*t* = −2.1, *p* = **0.04**
IL-1β mRNA (R)	1.0 ± 0.1	1.3 ± 0.1	*t* = 1.7, *p* = 0.1
IL-6 mRNA (R)	1.5 ± 0.1	1.1 ± 0.1	*t* = −2.5, *p* = **0.01**
IL-8 mRNA (R)	1.5 ± 0.3	1.0 ± 0.1	*t* = −1.6, *p* = 0.1
TNF-α mRNA (R)	1.6 ± 0.1	1.0 ± 0.1	*t* = −5.4, *p* < **0.001**
